# Colopleural fistula: Case report and review of the literature

**DOI:** 10.4103/1817-1737.41917

**Published:** 2008

**Authors:** A. Haleem A EI Hiday, Fahmi Y. Khan, Ahmed M. Almuzrakhshi, Hani EI Zeer, Fatima A. Rasul

**Affiliations:** *Hamad General Hospital, WCMC, Doha, Qatar*; 1 *Hamad General Hospital, Radiology Department, WCMC, Doha, Qatar*; 2 *Hamad General Hospital, Medicine Department, WCMC, Doha, Qatar*

**Keywords:** Colopleural fistula, colothorax fistula, fecal hydrothorax

## Abstract

We report a 28-year-old woman, pregnant, at 24 weeks, with 3-day history of right-sided chest pain and shortness of breath. Few hours after admission, she delivered a dead baby. She had a history of right partial hepatic lobotomy and cholecystectomy at UK on May 2004 because of multiple pyogenic liver abscesses. Chest examination revealed signs of hydrothorax on the right side. Chest X-ray showed pleural effusion on the right side. Pleural fluid was exudative with high neutrophils. Gram stain and culture showed multiple organisms. CT scan chest and abdomen with contrast, combined with barium enema, revealed right colothorax communication. Colothorax fistula was closed surgically. On the following days, the patient's symptoms resolved, and she was consequently discharged.

## Introduction

Fistulas are abnormal communications between two epithelial-lined surfaces. Gastrointestinal fistulas encompass all such connections that involve the alimentary tract, and they can be congenital or acquired in nature. Development of an acquired gastrointestinal (GI) fistula can greatly affect patient outcome, yet the clinical manifestations are often protean in nature; and the etiology, elusive. Acquired GI fistulas can be categorized as external or cutaneous if they communicate with the skin surface; or internal if they connect to another internal organ system or space, including elsewhere along the GI tract itself.[1] While traumatic diaphragmatic hernia is a well-recognized complication of blunt and penetrating injuries to the abdomen and thorax, strangulation of the large bowel that migrates through that hernia into the thorax with subsequent rupture and the development of fecal pneumothorax are most unusual.[2] Review of the literature revealed that only 8 cases[2–9] of colopleural fistula have been reported. Here we report a rare case of colopleural fistula in a 28-year-old woman who presented with shortness of breath.

## Case Report

A 28-year-old woman, pregnant, at 24 weeks' gestation was admitted from the Accident and Emergency Department, complaining of right-sided chest pain and shortness of breath of 3-day duration. Three days prior to admission, she developed sudden-onset, sharp, gradually and progressively increasing in severity right-sided chest pain radiating to the back, aggravated by breathing, with no relieving factors. This pain was associated with palpitation, vomiting, and difficult breathing. She denied having fever, weight loss, cough, abdominal symptoms, or trauma. Few hours after admission, she delivered a dead baby.

On April 2004, she developed right-sided exudative pleural effusion, for which she went to UK on May 2004, where she was found to have multiple pyogenic liver abscesses, for which she underwent right partial lobotomy and cholecystectomy.

On examination, the patient looked sick, tachypneic and distressed but not cyanosed, conscious, alert oriented. She was tachycardic but maintaining normal blood pressure. Chest examination revealed signs of hydrothorax on the right side.

**Figure 1:**
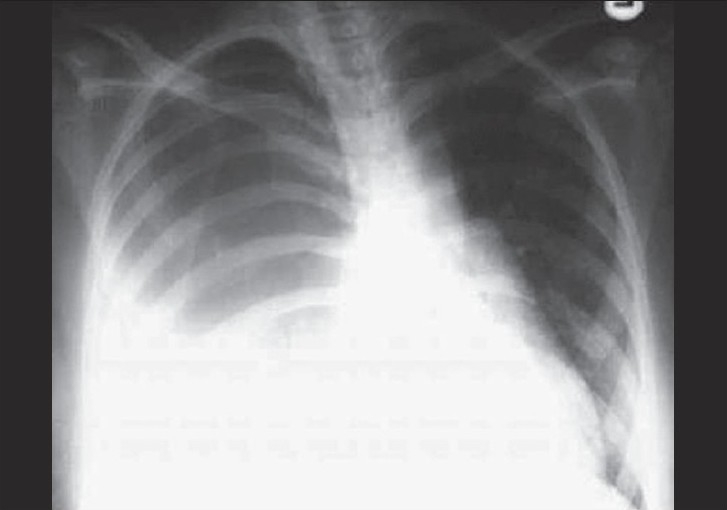
Chest X-ray shows homogenous opacity present at the middle and lower zones of the right lung obliterating the outline of the right dome of the diaphragm and the right CP angle suggesting pulmonary consolidation with large right pleural effusion. Left lung is clear

Investigations showed normal urea and electrolytes, slightly increased neutrophils with normal hemoglobin and platelets. Chest X-ray [Figure 1] showed absence of vascular marking along the right lung, as well as pleural effusion. Pleural fluid examination revealed high neutrophils, high protein, and very low glucose content. Gram stain and culture showed multiple organisms, profuse E. coli, Candida, and Pseudomonas Aeruginosa.

**Figure 2:**
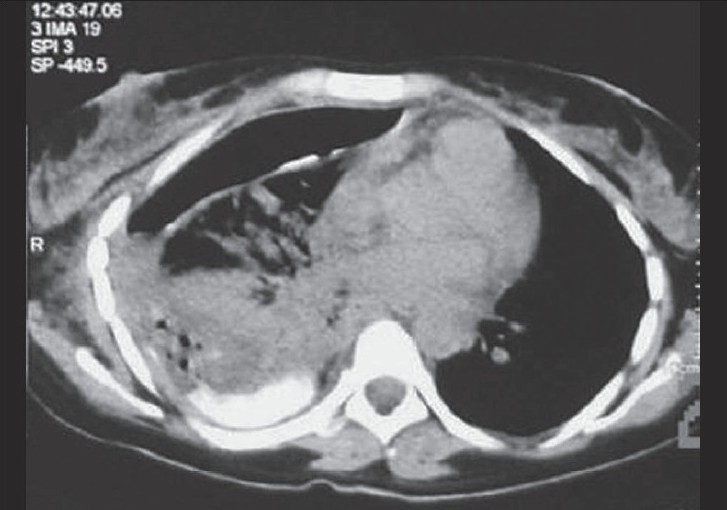
CT scan of the chest 1 day after barium enema shows barium layering along the posterior part of the right pleural cavity

CT scan of chest and abdomen showed collapsed right lower lobe with patent main bronchus and pleural effusion. CT scan chest and abdomen with contrast, combined with barium enema, revealed right colothorax communication [Figure 2].

On admission a chest tube was inserted, which drains a feculent odor, dirty fluid, as well as bubbles of air. Once the possibility of a colothorax communication was considered, surgeons were involved. Surgical notes revealed a herniated colon into right side of the thorax with a fistula between the hepatic flexure of colon and right thorax, confirmed by the histopathology of the resected segment, which revealed a fistula tract. On the following days, the patient became symptom free.

On follow-up evaluation approximately 4 weeks after the patient's discharge from the hospital, the patient was symptom free. Repeat physical examination was unremarkable. Chest X-ray and CT chest showed fluid-free chest.

## Discussion

The clinical presentation of traumatic diaphragmatic hernia is either acute or delayed in its appearance. Late presentations may occur months or years after the initial injury.[2,3]

Lacayo L. *et al.* reported the case of a 20-year-old woman who developed severe shortness of breath 4 hours after a cesarean section. Roentgenogram showed pleural effusion and tension pneumothorax; insertion of a chest tube drained liquid stool. She had sustained a left lower hemithorax stab wound 2 years prior to admission.[2] Seelig M. H. *et al.* reported a patient who, 2 years after being treated for a stab wound to the chest, presented with an acute tension fecopneumothorax caused by the incarceration of the large bowel in the thoracic cavity after an intrathoracic perforation.[4] On the other hand, our patient had undergone a major surgical intervention below the right diaphragm for multiple pyogenic liver abscesses, including right partial lobotomy and cholecystectomy, as well as draining right-sided pleural effusion almost 2 years before admission. These surgical manipulations may be the cause of a diaphragmatic defect leading to herniated colon, which ruptured.

Thus the patients in the above-mentioned cases, as our patient, presented with signs of right-sided hydrothorax; and when drained, the appearance of the material aspirated from the chest after placement of the chest tube, namely, its feculent odor and Gram stain that revealed a polymicrobial flora, raised the possibility of intestinal rupture. Although rare, colopleural fistulas present a diagnostic challenge, and delayed management can lead to increased morbidity.[3]

## Conclusion

Pleural fluid which yields unusual multiple organisms should raise the possibility of colopleural fistula, which usually develops in patients with underlying disorders or those who sustained surgical intervention under the diaphragm or stab wound to the chest; moreover, it should be kept in mind that colopleural fistula might develop as a delayed complication (years later) of traumatic diaphragmatic hernia.
